# A Fatal Cascade Following Endoscopic Retrograde Cholangiopancreaticography (ERCP) Complicated by Pancreatitis, Duodenal Perforation, Gastrointestinal Hemorrhage, and Multi-organ Failure

**DOI:** 10.7759/cureus.86983

**Published:** 2025-06-29

**Authors:** Sharath Rajagopalan, Vikash Kumar, Bharath P Bhushan, Leonard R Maier, Eric Huang

**Affiliations:** 1 Hospital Medicine, West Virginia University School of Medicine, Morgantown, USA; 2 Internal Medicine, West Virginia University School of Medicine, J.W. Ruby Memorial Hospital, Morgantown, USA; 3 Internal Medicine, West Virginia University School of Medicine, Morgantown, USA

**Keywords:** duodenal perforation, emergent surgery, interventional radiology guided embolization, : post ercp pancreatitis, pseudocyst of the pancreas

## Abstract

Endoscopic retrograde cholangiopancreatography (ERCP) is a valuable diagnostic and therapeutic procedure that carries inherent risks, with pancreatitis being a significant complication. While most cases resolve without major sequelae, severe complications can occur. A 65-year-old female with a history of pancreatitis developed post-ERCP pancreatitis following stent placement. The patient subsequently presented with walled-off fluid collections and experienced a catastrophic course complicated by duodenal perforation, severe hemorrhage, and multi-organ dysfunction, ultimately resulting in mortality. This case emphasizes the potential for life-threatening complications following post-ERCP pancreatitis and underscores the importance of vigilant monitoring and prompt intervention in managing these patients.

## Introduction

Endoscopic retrograde cholangiopancreatography (ERCP) is a widely used and essential diagnostic and therapeutic procedure for biliary and pancreatic diseases [1]. Post-ERCP pancreatitis is a well-recognized complication following ERCP, affecting around 3-10% of cases, with most cases being mild in severity and rather self-limiting; however, severe cases can lead to devastating outcomes including pancreatic necrosis, pseudocyst formation, systemic inflammatory response syndrome (SIRS), and multi-organ failure [2]. This report presents a case of post-ERCP pancreatitis that, despite early recognition and appropriate management of the post-ERCP complications, progressed rather quickly to the patient's eventual mortality. This case highlights the unpredictable nature of post-ERCP pancreatitis and underscores the need for continued research into improved preventive and therapeutic strategies to improve patient outcomes.

## Case presentation

**Figure 1:**
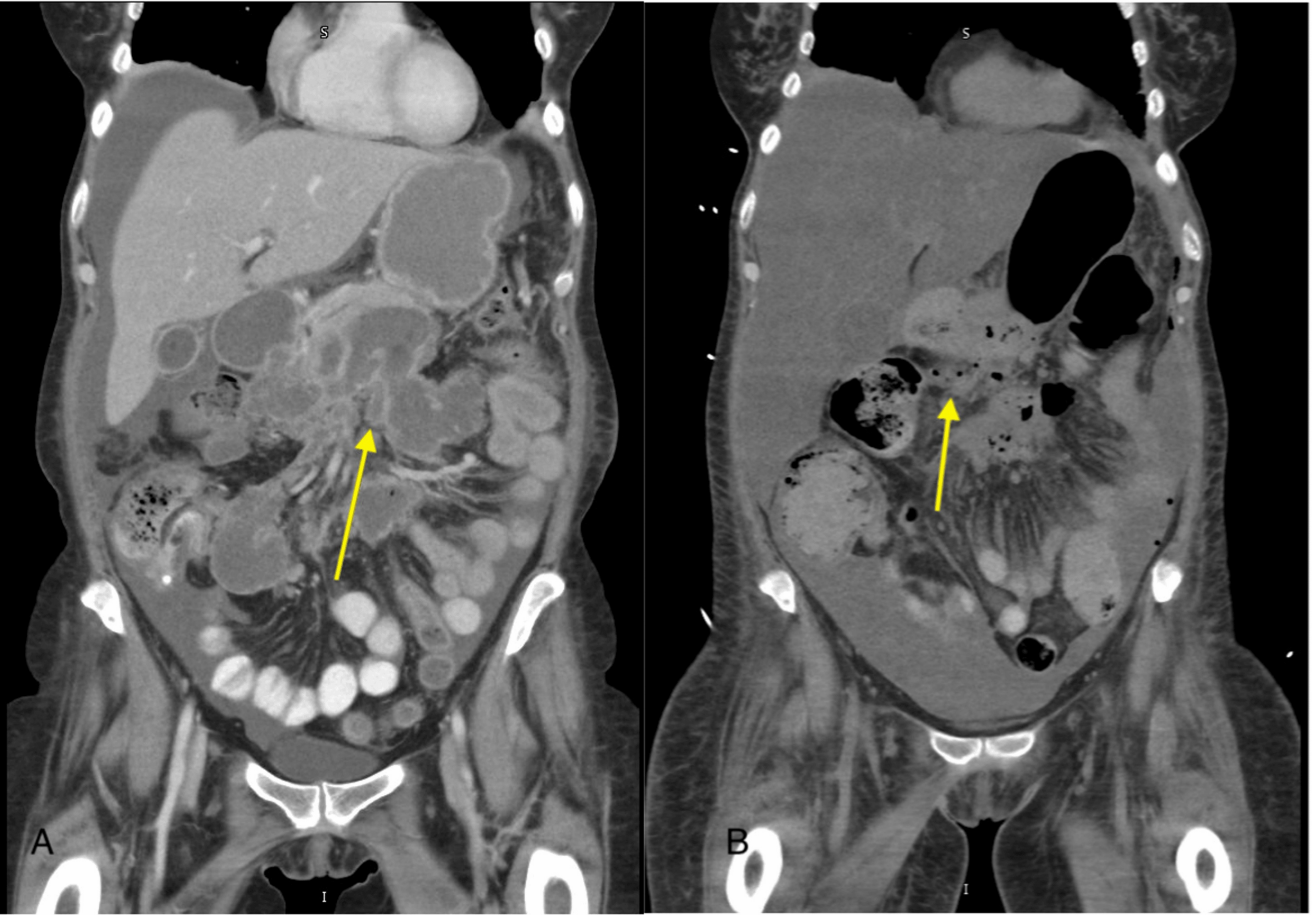
Initial CT of the abdomen and pelvis. (A) Coronal contrast-enhanced CT image of the abdomen demonstrating changes suggestive of suspected pancreatic pseudocyst (yellow arrow). (B) Coronal contrast-enhanced CT image of the abdomen demonstrating extra-luminal air (yellow arrow), indicating pneumoperitoneum likely due to perforation of a hollow viscus. CT: computed tomography

A 65-year-old female with a past medical history significant for a history of post-ERCP pancreatitis following stent placement two months prior to presentation and osteoporosis presented with likely recurrent pancreatitis and walled-off fluid collections. The patient had reported three weeks of abdominal pain, decreased oral intake, intermittent diarrhea for the past two months, along with 10-pound weight loss. Initial lipase level on presentation was reported to be 13 U/L (reference range: 10-80 U/L). Initial computed tomography (CT) of the abdomen and pelvis revealed sequelae of pancreatitis with multiple loculated collections surrounding the pancreas, extending to the bilateral pararenal space, suggesting likely pseudocyst based on imaging results (Figure 1, panel A).

Although the serum lipase level on presentation was within the normal reference range, this was considered not uncommon in subacute or recurrent cases of pancreatitis, especially in the context of this patient's recent prior episode of pancreatitis, timeline of symptoms, and the presence of a likely pseudocyst noted on imaging. Upon admission, the patient was started on standard treatment protocols for presumed acute-on-chronic recurrent pancreatitis, including aggressive intravenous fluid resuscitation and close monitoring of vital signs and urine output. Given the significant fluid collections, including large-volume ascites noted on imaging, therapeutic paracentesis was performed on hospital day three, resulting in the drainage of approximately 3 L of fluid. The procedure was undertaken both for diagnostic purposes to evaluate for potential pancreatic duct leak and symptomatic relief.

Despite these initial interventions, the patient's clinical status began to deteriorate rapidly. The patient developed severe, intractable abdominal pain and subsequently sustained a fall. A CT scan at this time revealed an ominous finding of new-onset pneumoperitoneum, suggesting hollow viscus perforation (Figure 1, panel B). Concurrent laboratory studies demonstrated significant systemic inflammatory response, with marked leukocytosis with a white block cell count of 22,000 cells/μL (reference range: 3.5-11 x 10³ cells/μL) and an elevated serum lactate level of 10.1 mmol/L (reference range: 0-1.3 mmol/L), indicating likely tissue hypoperfusion and systemic inflammatory response and likely early shock state.

Given these concerning findings, the patient was emergently transferred to the intensive care unit for higher-level care. The patient’s condition rapidly progressed to frank hemodynamic instability, necessitating immediate surgical exploration. Emergency laparotomy revealed two critical findings as follows: a perforation in the fourth portion of the duodenum (D4) and active hemorrhage from the gastroduodenal artery (GDA). The severity of bleeding necessitated activation of the massive transfusion protocol, during which the patient received eight units of packed red blood cells, three units of fresh-frozen plasma, two ampules of sodium bicarbonate, and 2,000 mg of calcium to maintain hemodynamic stability and address coagulopathy.

**Figure 2:**
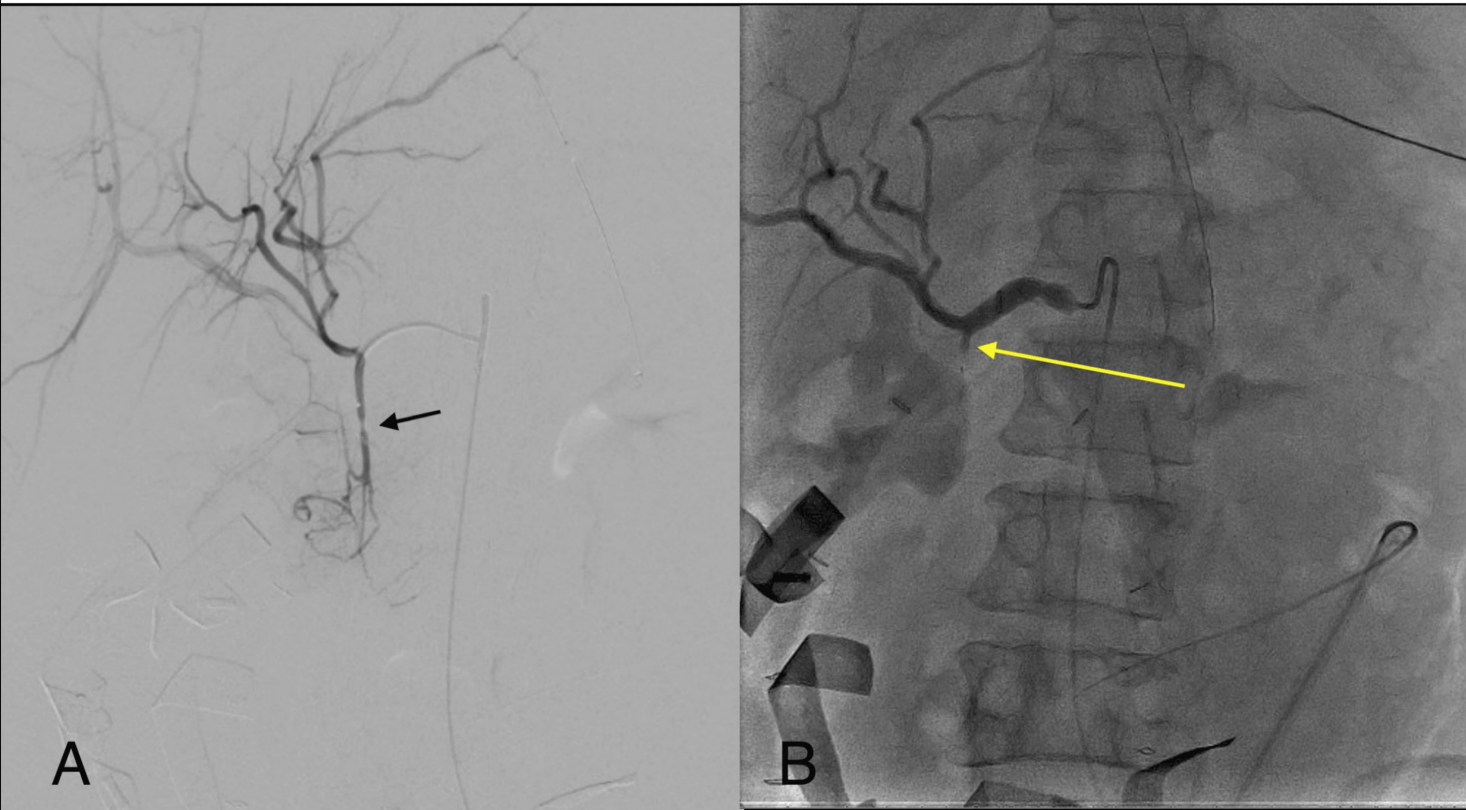
Angiographic embolization of the gastroduodenal artery following post-operative bleeding. (A) An image of an angiogram showing the hepatic arterial system, with the black arrow indicating the presumed focal irregularity of the gastroduodenal artery, prior to intervention. (B) An image of an angiogram showing the hepatic arterial system following embolization of the gastroduodenal artery (yellow arrow) using a 3-5 mm microvascular plug.

Following the initial surgical intervention, ongoing bleeding necessitated urgent interventional radiology consultation for angiographic embolization of the gastroduodenal artery (Figure 2, panels A and B). Despite these aggressive interventions, the patient's condition continued to deteriorate.

The patient developed refractory shock requiring multiple vasopressors (including norepinephrine and vasopressin) and mechanical ventilation for respiratory support. Most concerning was the patient’s persistent severe anemia, with hemoglobin levels dropping precipitously from 5.0 g/dL to 3.0 g/dL (reference range: 11.2-15.2 g/dL) despite aggressive blood product replacement, suggesting ongoing blood loss or consumption.

As the patient's multi-organ dysfunction progressed despite maximal medical and surgical intervention, a family conference was convened to discuss goals of care. After careful consideration of the patient's previously expressed wishes regarding end-of-life care, and in light of the patient’s grim prognosis, the patient’s husband decided to transition the patient to comfort measures only (CMO) protocol, and the patient expired shortly thereafter.

## Discussion

Endoscopic retrograde cholangiopancreatography (ERCP) is an important gastrointestinal procedure being utilized for a range of conditions, including obstructive jaundice, choledocholithiasis, acute cholangitis, biliary stricture, and malignant biliary obstruction. The procedure has been performed at a steadily increasing rate over the last decade, with an estimated 350,000-500,000 cases annually in the United States [3]. Though several factors, including patient age and comorbidities (including malignancy), contribute to outcomes, some have estimated a mortality rate of nearly 5% at 30 days and 11.9% at three months from procedure [4]. Complications from ERCP, which can prove fatal, include post-ERCP pancreatitis, unresolved cholangitis, post-sphincterotomy bleeding, bile leak, and duodenal perforation. Rate of post-ERCP pancreatitis is estimated to be nearly 2.1% and the mortality rate is nearly 0.2% [5]. Duodenal perforation occurs during an estimated 1% of ERCP cases [6]. Although serum lipase level on presentation was within the normal reference range, this was felt to not be uncommon in subacute or recurrent cases of pancreatitis, especially in the context of this patient's prior recent episode of pancreatitis, timeline of symptoms, and presence of likely pseudocyst noted on imaging, which usually requires more then four weeks after the initial episode of acute pancreatitis for maturation [7,8]. Post-ERCP pancreatitis is either diagnosed with an amylase or lipase trending to ≥3 times the upper-limit-of-normal after procedure, or as per the Atlanta classification of acute pancreatitis [8].

Measures to prevent post-ERCP pancreatitis include avoiding unnecessary ERCP, administering non-steroidal anti-inflammatory drugs (NSAIDs) when not contraindicated, and using intravenous fluids cautiously to prevent volume overload in high-risk patients (e.g., those with congestive heart failure, decompensated cirrhosis, or advanced chronic kidney disease). Additional preventive strategies include wire-guided biliary cannulation, employing techniques that minimize inadvertent pancreatic duct cannulation (such as the double guidewire technique or transpancreatic biliary septotomy), and, in high-risk patients, consideration of prophylactic pancreatic duct stenting [5]. Management of post-ERCP pancreatitis, similar to other forms of acute pancreatitis, consists of analgesia, supportive care, and intravenous fluid administration.

Post-ERCP duodenal perforations are subdivided into four types via the Stapfer et al. classification system based on location [9]. Type 1 perforation occurs as a result of pressure from the endoscope during sweep at the relatively thin medial or lateral wall of duodenum. Type 2 perforation occurs as a result of sphincterotomy or pre-cut needle-knife at periampullary region of the duodenum. Type 3 perforation occurs during cannulation of the pancreatic duct, typically from guidewire piercing a side branch. Type 4 micro-perforations occur at the retroperitoneum and are suspected to be the result of compressed air used to maintain the lumen open during endoscopy [9]. In this patient's case, based on the exploratory laparotomy identifying perforation at the fourth portion of the duodenum, type 1 perforation would be suspected if it was sustained during the ERCP procedure. In this patient, due to the relatively delayed onset of decline from the time of the ERCP procedure, the perforation may have been exacerbated by inflammatory changes associated with post-ERCP pancreatitis and pseudocysts. Risk factors for perforation include sphincterotomy, deep incisions, guidewire perforation, sphincter of Oddi dysfunction, older age, and prolonged procedures [9].

Early identification of duodenal perforation is imperative to avoiding fatal outcomes. Only an estimated 27% of duodenal perforations are identified during the ERCP (with or without direct visualization) and confirmed with gastrografin administration and concurrent upper gastrointestinal imaging for contrast extravasation [10]. In such cases, endoscopic management options include suturing or placement of clips or intra-ductal stent; however, if significant extravasation is present, then surgical management may still be required ranging from perforation repair and choledochotomy, choledochojejunostomy, or pancreatoduodenectomy, depending on the extent [6]. After ERCP, computed tomography (CT) and serial abdominal examination are also important diagnostics [11].

## Conclusions

This case underscores the potential catastrophic and life-threatening complications following ERCP, and demonstrates the swift clinical deterioration even with appropriate interventions. It reinforces the need for close monitoring in high-risk individuals and maintaining a high index of suspicion for early signs of post-procedural complications following routine procedures. Post-ERCP complications, such as perforation, hemorrhage, and severe pancreatitis, are associated with inflammatory cascades that progress swiftly despite appropriate management. This case reinforces the need to focus on preventive techniques during procedures to mitigate and possibly prevent post-ERCP complications. Increased awareness and close monitoring in the peri- and post-procedural states, along with a coordinated interdisciplinary team approach, are essential in reducing morbidity and mortality and improving patient outcomes following procedural interventions.
